# Polymorphous adenocarcinoma of minor salivary gland: Case presentation and literature review

**DOI:** 10.1016/j.ijscr.2024.109647

**Published:** 2024-04-28

**Authors:** Hassan Alhazzani, Rafeef Ibrahim Alhajress, Abdulrahman Alghulikah, Somaya Alabaishi, Mohammad Saeed Algarni, Ahmad A. Altuwaijri

**Affiliations:** a College of Medicine, King Saud University, Riyadh, Saudi Arabia; b Otolaryngology - Head & Neck Surgery, King Abdulaziz University Hospital, Riyadh, Saudi Arabia; c ENT Department, Security Forces Hospital, Riyadh, Saudi Arabia; d Department of Anatomical Pathology, Security Forces Hospital, Riyadh, Saudi Arabia; e Otolaryngology, Head & Neck Surgery and Microvascular Reconstructive Surgery, Saudi Arabia; f Department of Otolaryngology-Head and Neck Surgery, Security Forces Hospital, Riyadh, Saudi Arabia

**Keywords:** Polumorphous adenocarcinoma, Salivary gland tumor, Minor salivary gland, Nasopharynx, Malignancy, Case report

## Abstract

**Introduction and importance:**

Polymorphous low-grade adenocarcinoma (PLGA) is a rare neoplasm arising from minor salivary glands, representing approximately 3 % of head and neck tumors. The clinical presentation of PLGA is defined as a painless, slow-growing tumor, mostly occurring in the palate. We report a case of PLGA with a rare presentation.

**Case presentation:**

A 76-year-old male, known case of hepatitis B, diabetes, and hypertension, presented to the emergency department complaining of spitting blood and dysphagia. Imaging showed a heterogeneous enlarged left tonsil with hyperemia of the mucosa, and air foci. Biopsy with excisional biopsy confirmed the diagnosis of PLGA. The patient underwent completion tonsillectomy and selective neck dissection which yielded tonsillar tissue with underlying PLGA, and reactive lymph nodes with no malignant tissue respectively, margins were negative for malignancy.

**Clinical discussion:**

Polymorphous low-grade adenocarcinoma is a rare lesion with clinical behavior resembling that of a benign neoplasm. Predominantly occurring in the oral cavity, especially on the hard palate, buccal mucosa, and retromolar region, with fewer cases in the upper lip. Occurrence in the nasopharynx and oropharynx is rare. PLGA presents as painless slow-growing masses, typically in females aged 50–60. Local excision with careful margin evaluation is the preferred treatment, with good prognosis compared to other carcinomas.

**Conclusion:**

PLGA is rare, with limited reported case from around the world. It is mostly seen in adults between their fifth and sixth decades with female predominance. PLGA is diagnosed using imaging, immunohistochemistry. Owing to the limited cases there is no standard approach to treating PLGA. However, most cases are managed with local excision and showed an excellent response in terms of tumor nonrecurrence.

## Highlights

• Polymorphous low-grade adenocarcinoma is rare minor salivary gland tumor.
• This case of polymorphous low-grade adenocarcinoma that presented only with a painless tonsillar mass.
• This is the first reported case in the Middle East region.

## 1 Introduction

Polymorphous low-grade adenocarcinoma (PLGA) is a rare minor salivary gland tumor with variable presentations. It has been previously described as “lobular carcinoma” and “terminal duct carcinoma”. Freedman and Lumerman [1] and Batsakis et al. [2] simultaneously described it as such due to histopathologic characterization and polymorphic attribution similar to lobular carcinoma and terminal duct carcinoma. In 1984 Evan [3] described the PLGA in recognition of its slow local aggressiveness which is recognized in World Health Organization (WHO) until today. Minor salivary gland tumors are responsible for 2–4 % of head and neck tumors [4]. Castel et al. [5] described that complete surgical excision along a series of 164 cases is an appropriate therapy. Evan et al. [3] approached multiple cases with complete excision followed by iridium implant and radiotherapy, with both approaches having comparable outcomes.

We present a case of polymorphous low-grade adenocarcinoma that presented with spitting blood and a painless tonsillar mass and was treated with complete surgical excision with adequate hemostasis. To the best of our knowledge this first reported case in the Middle East region.

A literature search was done using Web of Science, PubMed, Google Scholar, and the work has been reported in line with the SCARE criteria [6]. The Articles were reviewed to extract information on the PLGA presentation, diagnosis, management options and the outcomes.

## 2 Case presentation

**Fig. 1:**
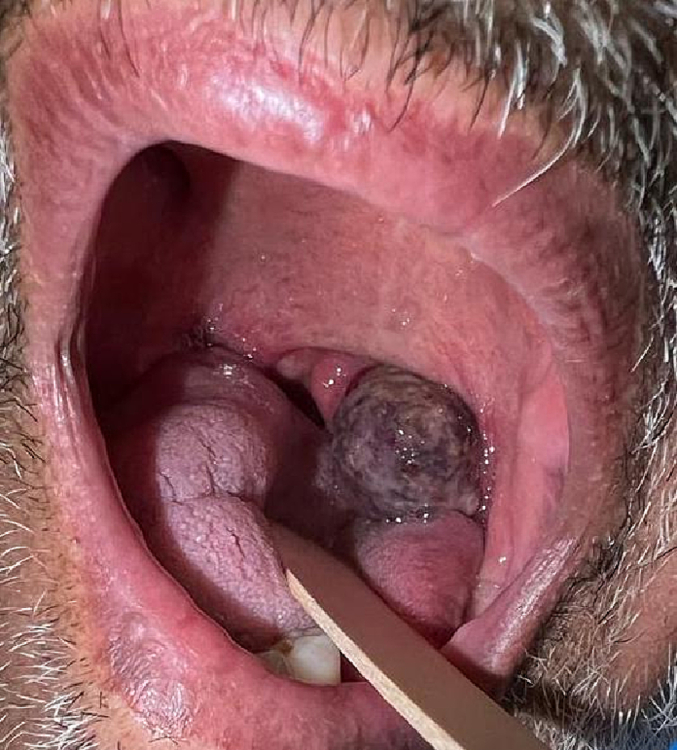
Oral examination showed a left dark grade 4 tonsillar mass pushing the uvula to the right with overlaying mucosa appearing bluish.

**Fig. 2:**
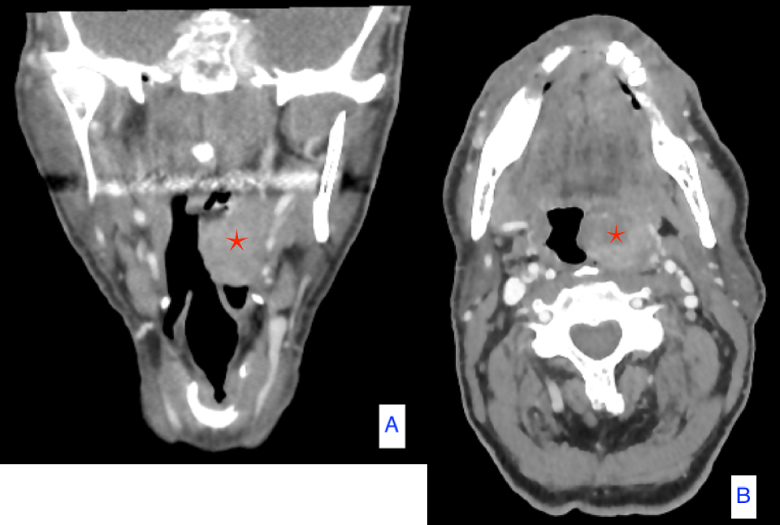
Computed tomography scans with contrast of the tonsillar mass (red asterisk) that occupies the left side of oropharynx. (For interpretation of the references to colour in this figure legend, the reader is referred to the web version of this article.)

A 76-years-old male, non-smoker, known case of Hepatitis B, Diabetes, and hypertension with a history of an old stroke, presented to the emergency department in January 2023 complaining of 3 days history of spitting blood, associated with dysphagia for the past 7 days. He reported no constitutional symptoms. The head and neck examination showed a left dark firm grade 4 tonsillar mass pushing the uvula to the right side with the overlying mucosa appearing erythematous and normal in texture. The swelling was moderately firm and no tenderness with multiple bleeding points (Fig. 1). There were no other abnormalities found on physical examination. A flexible fiberoptic scope was done which showed left palatine tonsillar enlargement reaching supraglottic region with true vocal fold movement bilaterally. A contrasted Computed Tomography (CT) scan of the neck showed a 2.7 × 2.5 × 3.4 cm heterogeneous enlarged left tonsillar occupying half of the oral part of pharynx with hyperemia of the mucosa, and air foci without collection (Fig. 2). The metastatic workup, which included chest radiographs, Computed Tomography (CT) scan of chest, abdomen, and pelvis, and head and neck magnetic resonance imaging (MRI), was negative.

**Fig. 3:**
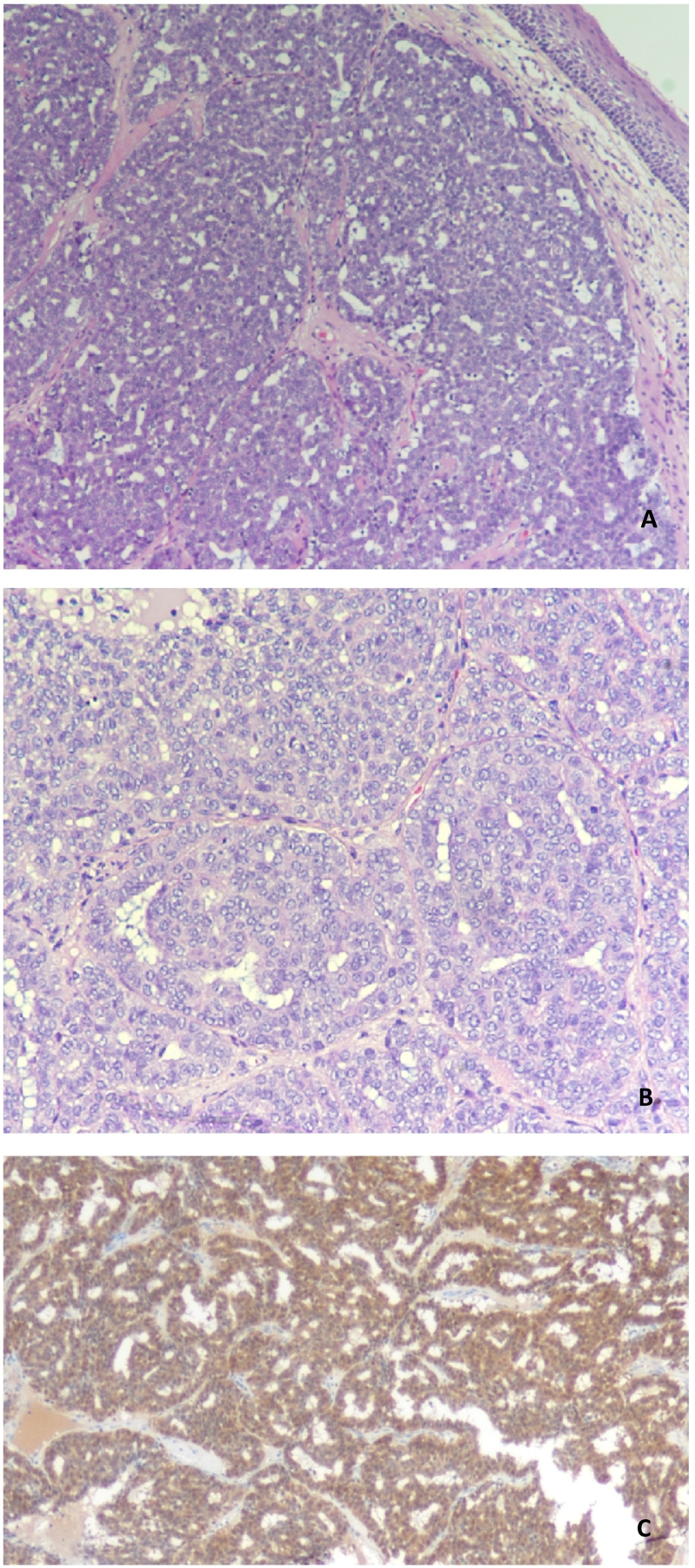
Photomicrographs of the examined biopsy showing (A) an infiltrative sub-epithelial lesion; (B) at high power magnification show the tumor cells are round to oval. With pale nuclei; (C) S100 immunostaining show diffuse positive staining.

An excisional biopsy of the mass was carried out which confirmed diagnosis of Polymorphous Adenocarcinoma of Minor Salivary Gland. Histopathological examination revealed the following characteristic, sub-epithelial infiltrative lesion that shows different patterns of arrangement include solid, cribriform, duct and tubules (Fig. 3A). The tumor cells are round to oval with pale nuclei and vesicular chromatin and inconspicuous nucleoli (Fig. 3B). Mitotic activity was very low. S100 immunohistochemical stain (IHC) was performed and found to have strong positive staining (Fig. 3C). The overall features were suggestive of polymorphous adenocarcinoma.

Patient was staged after complete metastatic work up as T2N0M0 Stage 2. Case was discussed in tumor board; a decision was made to go for selective neck dissection left level II and III with completion left tonsillectomy. Intra operative frozen section came back negative for all margins. Histopathology of neck dissection came negative for malignancy.

Patient made an uneventful recovery. Follow up 2 weeks, 3 months and 6 months post operatively showed good healing with no signs of clinical recurrence.

## 3 Discussion

Polymorphous low-grade adenocarcinoma is a rare lesion with a clinical behavior similar to that of a benign neoplasm. The majority of PLGA of minor salivary glands cases arise in the oral cavity (90 %) more commonly occurring in the hard palate followed by the buccal mucosa, and retromolar region. The remaining extraoral cases are mostly seen in the upper lip (10 %). Nasopharynx locations have been reported to be less than 0.5–1 % of the cases [7]. The occurrence of PLGA in the minor salivary glands of the oropharynx is rare, occasionally seen in the soft palate, base of the tongue, and the tonsils [[8], [9], [10]]. Only two previous cases of PLGA in the tonsils were reported in the literature [10], this is the third reported case in the literature.

PLGA has a wide spectrum of clinical presentations which defined as painless slow-growing mass, coated by non-ulcerated mucosa, and may occurred in different sizes ranged between 1 and 4 cm [11]. It has been reported in many studies and clarified to be a female predilection, tend to be found in the fifth and sixth decades.

PLGA appears in various morphology, as a sequence it may be misdiagnosed with monomorphic adenoma or pleomorphic adenoma; a type of benign salivary gland neoplasm. Although, PLGA may be misdiagnosed with malignant mixed tumor, adenoid cystic carcinoma, or adenocarcinoma. Hence, immunohistochemistry has been suggested in many studies for its ability to differentiate PLGA from pleomorphic adenoma and adenoid cystic carcinoma.

Evans et al. conducted a study of PLGA among 40 cases with ten years follow up concluded a significant statistical correlation between focal papillary growth and cervical lymph nodes, and between positive or unknown surgical margins and local recurrence, moreover, local recurrence appeared in thirteen patients, and the study showed low rate of distant metastasis appeared in three cases [12].

The most effective therapy is the local excision with the importance of evaluating surgical margins [13]. Radiation and chemotherapy may not be required in the postoperative phase. The prognosis considered to be good when compared to adenoid cystic and mucoepidermoid carcinoma [14].

## 4 Conclusion

PLGA is rare, with limited reported case from around the world. It is mostly seen in adults between their fifth and sixth decades with female predominance. PLGA is diagnosed using imaging, immunohistochemistry. Owing to the limited cases there is no standard approach to treating PLGA. However, most cases are managed with local excision and showed an excellent response in terms of tumor nonrecurrence.

## Consent

Written informed consent was obtained from the patient for publication of this case report and accompanying images. A copy of the written consent is available for review by the Editor-in-Chief of this journal on request.

## Ethical approval

Ethical approval was not required for this case report as it involves the description of an individual patient case and does not constitute research involving interventions or experiments.

## Funding

No funding was received for this study.

## Author contribution

Hassan Alhazzani: Analysis and interpretation of data, drafting the article, final approval of the version to be submitted.

Rafeef Ibrahim Alhajress: Analysis and interpretation of data, drafting the article, final approval of the version to be submitted.

Abdulrahman Alghulikah: Analysis and interpretation of data, drafting the article, final approval of the version to be submitted.

Somaya Alabaishi: Analysis and interpretation of data, drafting the article, final approval of the version to be submitted.

Mohammed Saeed Algarni: Analysis and interpretation of data, drafting the article, final approval of the version to be submitted.

Ahmad A Altuwaijri: Analysis and interpretation of data, drafting the article, final approval of the version to be submitted.

## Guarantor

Hassan Alhazzani

Rafeef Ibrahim Alhajress

Abdulrahman Alghulikah

## Conflict of interest statement

The authors declare that they have no conflicts of interest.

## Data Availability

The data used to support the findings of this study are included within the article.
